# Haplotype-based membership inference from summary genomic data

**DOI:** 10.1093/bioinformatics/btab305

**Published:** 2021-07-12

**Authors:** Diyue Bu, Xiaofeng Wang, Haixu Tang

**Affiliations:** Department of Informatics, Luddy School of Informatics, Computing, and Engineering, Indiana University, Bloomington, IN 47408, USA; Department of Informatics, Luddy School of Informatics, Computing, and Engineering, Indiana University, Bloomington, IN 47408, USA; Department of Informatics, Luddy School of Informatics, Computing, and Engineering, Indiana University, Bloomington, IN 47408, USA

## Abstract

**Motivation:**

The availability of human genomic data, together with the enhanced capacity to process them, is leading to transformative technological advances in biomedical science and engineering. However, the public dissemination of such data has been difficult due to privacy concerns. Specifically, it has been shown that the presence of a human subject in a case group can be inferred from the shared summary statistics of the group, e.g. the allele frequencies, or even the presence/absence of genetic variants (e.g. shared by the Beacon project) in the group. These methods rely on the availability of the *target’s genome*, i.e. the DNA profile of a target human subject, and thus are often referred to as the *membership inference* method.

**Results:**

In this article, we demonstrate the *haplotypes*, i.e. the sequence of single nucleotide variations (SNVs) showing strong genetic linkages in human genome databases, may be inferred from the summary of genomic data without using a target’s genome. Furthermore, novel haplotypes that did not appear in the database may be reconstructed solely from the allele frequencies from genomic datasets. These reconstructed haplotypes can be used for a haplotype-based membership inference algorithm to identify target subjects in a case group with greater power than existing methods based on SNVs.

**Availability and implementation:**

The implementation of the membership inference algorithms is available at https://github.com/diybu/Haplotype-based-membership-inferences.

## 1 Introduction

Fueled by the rapid advance of the genome sequencing technologies (Shendure et al., 2017)and their applications, human genome data have been extensively collected and disseminated to facilitate human genome studies (HGS) (Ansorge, 2016). In particular, an increasing number of projects [e.g. the 1000 genomes project (GenomeAsia100K Consortium, 2015), the personal genome project (Church, 2005), the UK Biobank project (Bycroft et al., 2017) and the GenomeAsia 100 K Project (GenomeAsia100K Consortium, 2019)] aim to collect the genomic sequences along with phenotypic and health-related information from a large cohort (up to a million) of disease patients and healthy human subjects, providing invaluable resources for biomedical research, such as genome-wide association study (GWAS) of human diseases and clinical conditions. However, the access and sharing such data are sometimes limited due to privacy concerns [e.g. as guided by the National Institutes of Health (NIH) Genomic Data Sharing (GDS) Policy (National Institutes of Health Genomic Data Sharing Governance Committees, 2014)], because it is well known that human genomic data contain personal identifiable information that may impose potential privacy threats to the confidentiality of participants of HGS, especially when the genomic data are linked to participants’ clinical records.

More striking results come from the previous studies referred to as the *membership inference* or *re-identification* attack (Erlich and Narayanan, 2014), which demonstrate that the presence of a human subject in a large human genome database could be inferred from the summary statistics of genomic variations in the database that are often shared in GWAS research, if the genetic profile of the target was known. For instances, Homer et al. (2008) shows even when the database contains thousands of human genomes, a human subject in the database can be re-identified with high confidence from the *allele frequencies* on thousands of SNVs. Follow-up researches also show the improved statistical methods for membership inference (Sankararaman et al., 2009), as well as the re-identification risks in other types of summary statistics in genomic studies (e.g. linkage equilibrium, gene expression levels, etc.), particularly when they are linked with other phenotypic and clinical data (Deznabi et al., 2018; Gymrek et al., 2013; Harmanci and Gerstein, 2016; Humbert et al., 2013; Lippert et al., 2017; Wang et al., 2009).

Notably, membership inference can succeed even by simply using the presence/absence (instead of the allele frequencies) of genetic variants through the query to a genome database as enabled by a Beacon service (Cupak, 2016). The Global Alliance for Genomics and Health (GA4GH), which serves as a global platform for responsible genomic and health data sharing with consistent policy, standards and protocols (Page et al., 2016), initiates the Beacon project (Cupak, 2016) for disseminating human genomic data by allowing users (i.e. biomedical researchers) to query if a specific genetic variant is present in a set of human genome database, each operated by an independent institute, through a unified web service platform. The queries accepted by the Beacon services follow the forms like ‘Do you have any genomes with nucleotide A at position 123 457 on chromosome 2?’, while the responses from Beacon would be ‘Yes’ or ‘No’ (True/False answer). The users can therefore collect the information: if the queried variant can be found in any database registered at Beacon. None of additional statistics (such as actual counts of variants) is exposed to the users. Only very limited summary of genomics data is shared by Beacon for privacy protection purpose, while still informative for researchers: they can contact the respective data owners to request full access to genome databases that contain the genomes carrying the variants of their interests (Cupak, 2016). However, Shringarpure and Bustamante (Shringarpure and Bustamante, 2015) devise a likelihood-ratio test (LRT) that can re-identify a target human subject in a Beacon database based on merely the presence/absence information of single-nucleotide variants (SNVs) through repeatedly Beacon queries and the known genomic profile of the subject. Further researches show that by utilizing additional information from public data, the membership inference can be improved through Beacon queries, e.g. by using the optimal attack introduced by Raisaro et al. (2017) that considers the queries of the minor allele frequencies (MAFs). On the other hand, the defense strategies techniques (Al Aziz et al., 2017; Ayoz et al., 2020b; Bu et al., 2018; Raisaro *et al.*, 2017; Wan et al., 2017) are also proposed against the membership inference attacks on Beacon services, e.g. by rejecting a proportion of queries if their answers were considered to leak sufficient information of a potential target in the database (Al Aziz *et al.*, 2017; Bu *et al.*, 2018; Raisaro *et al.*, 2017; Wan *et al.*, 2017) or by including the genomes of relatives in the database (Ayoz *et al.*, 2020b).

Thus far, most of the membership inference methods on human genome database are based on SNVs, and assume different SNVs are independent. Wang *et al.* (2009) propose several methods to enhance Homer’s SNV-based approach (Homer *et al.*, 2008) by taking as input the linkage disequilibrium (LD) among SNVs in addition to the frequency table. Exploiting the information in LDs and the correlations among SNVs, new test statistics are introduced with a higher power than the Homer’s method. Deznabi *et al.* (2018) develops a Markov chain-based recombination model between haploblocks, along with the phenotype and kinship information, to infer the hidden part of an individual’s genomes from the publicly shared partial genomic sequences. Von Thenen et al. (2019) applies the Markov chain model and LD to infer the membership and the hidden part of the target’s genome from Beacon tables. More recently, Ayoz et al. (2020a) shows that a target’s genome can be reconstructed through the differential analyses of the shared Beacon tables before and after the target’s genome is added to an existing private genome database.

One limitation of the current membership inference methods is that they require a target’s genome, i.e. the known genetic profile of the target human subject. From the privacy protection perspective, one may argue that obtaining the target’s genome is already a privacy breaching of the target human subject, which may impose much more severe harm to the target human subject than a membership inference attack. In practice, it is not straightforward to obtain the target’s genome, and thus the actual risk of the membership inference attack is considerably low. As a balance between the benefit and risk of human genomic data sharing, NIH has updated its GWAS data sharing policy in 2019, which now allows the summary results from most HGS (except those ‘sensitive studies’) to be shared broadly.

In this article, we present a novel membership inference approach, aiming to infer the presence of a *haplotype*, i.e. a sequence of strongly linked genetic variants in a human genome database based solely on the summary statistics (e.g. allele frequencies or the presence/absence of individual variants) without using a target’s genome. We demonstrate that the haplotype-based membership inference can:

- be performed without the knowledge of the target’s genome, which is required by previous approaches (Al Aziz *et al.*, 2017; Bu *et al.*, 2018; Homer *et al.*, 2008; Raisaro *et al.*, 2017; Shringarpure and Bustamante, 2015; von Thenen *et al.*, 2019; Wan *et al.*, 2017; Wang et al., 2017).
- achieve high confidence of the presence of rare haplotypes in a database, i.e. with the very low frequency in the general population (Note that for those relatively common haplotype, their probability to be present in a reasonable size genome database is high even without membership inference. However, this does not provide much identifiable information about the individuals in the database because a substantial fraction of general population carry this haplotype anyway. On the other hand, the rare haplotypes, which we aim at in this article, have very low frequency in the general population, and thus each of them is almost sufficient to identify a human subject (or her close biological relatives) in a target human genome database.).
- improve the power of the membership inference using the inferred haplotypes when a target’s genome is indeed available.
- reconstruct novel haplotypes in a genome database based on its summary statistics that have not been observed in advance (e.g. in public human genome database).

We consider the applications of the haplotype-based membership inference method to two forms of summary statistics as input, of which each is viewed as a set of returned answers of the queries to a (private) human genome database, i.e. when a user queries a particular target allele at a specific variation site in the human genome, the database will answer

- the frequency of the *target allele* (herein denoted as *frequency table*); or
- whether the *target allele* is present in the database (herein denoted as *Beacon table*).

Apparently, the frequency table is a more general form and contains more information than the Beacon table.

## 2 Materials and methods

**Fig. 1.**
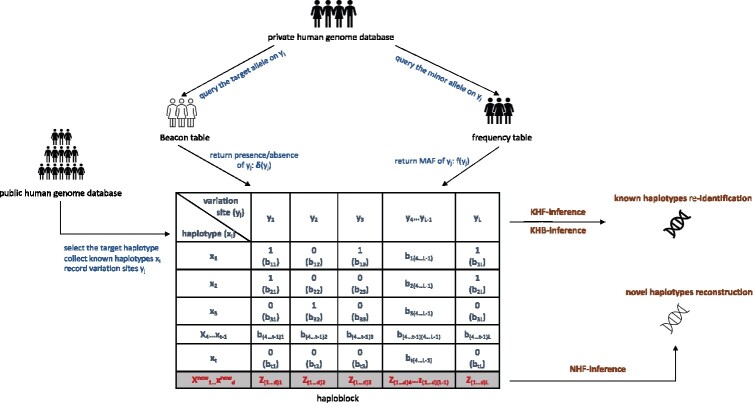
The workflow of haplotype-based membership inferences. Similarly with the SNV-based inferences, the inference is performed on either the frequency table (by KHF-inference) or the Beacon table (by KHB-inference), both of which are derived from a private genome database comprising *N* genomes (2 *N* haplotypes) and may be published or shared through queries to the database. However, in both cases, the haplotype-based inferences do not rely on the target’s genome. Instead, they aim to detect a target haplotype that is rare (with low frequency) in the general population. The target haplotypes are selected based on a public human genome database. When the frequency table is available, the NHF-inference algorithm is introduced to reconstruct a novel haplotype that was not even observed in the public database. Notably, all three haplotype-based algorithms presented here consider the constraints on the counts of all putative *t* haplotypes (observed in the public database) within the target haploblock (illustrated table at the center of the figure), imposed by the frequencies or the presence/absence of the minor allele at each variation site (see texts for details)

Most existing membership inference methods combine the weak statistical power provided by thousands of individual SNVs through the differential analysis of summary statistics from the genome database containing a target human subject versus those from the database without the target. Our approach, instead, starts from the *haplotypes*, as a sequence of strongly linked SNVs. Because of the LD structure, the human genome, can be partitioned into consecutive segments, i.e. haploblocks, so that the SNVs within the same haploblock show the strong linkage while the SNVs across the haploblock are only weakly linked. Each haploblock may contain dozens to hundreds of SNVs depending on the threshold of LD to determine their boundaries. Because of the strong correlation among SNVs with the haploblocks, only a small number (e.g. < 100) out of $2^{L}$ allelic sequences over the *L* SNVs in a haploblock can be observed, each with a different frequency in the human population. Therefore, the haplotype-based membership inference has two advantages comparing with the SNV-based inference (Fig. 1): (i) the statistical power of a haplotype is stronger than the combined power of the individual SNVs in haplotype, and thus the haplotype-based inference may achieve a higher power than the SNV-based inference given the same frequency or Beacon table; and (ii) we may estimate the probability of each haplotype to be present in a genome database based on the frequency table or the Beacon table from the database, which does not require the knowledge of a target’s genome. Finally, the haploblock structure in human genome has been well established based on the human genomes from population genomics project such as the 1000 Genomes project (GenomeAsia100K Consortium, 2015). The haplotypes in these haploblocks can be retrieved using the software tools such as HaploView (Barrett et al., 2005), which can be used in the haploblock-based inference as described below.

Given a frequency or Beacon table over the SNVs in a private genome database, the haplotype-based membership inference attempts to evaluate if a haplotype retrieved is carried by any genome in the database. We devise the haplotype-based inference methods for three scenarios depending on the input data (frequency versus Beacon table) and the assumption of the haplotype (known versus novel). In the first two scenarios, we consider the haplotype to be tested is known (and can be retrieved from public genomes). We build two inference algorithms for the input of *frequency table* (denoted as KHF-inference, representing the Known Haplotype Inference on *frequency table*) and the *Beacon table* (denoted as KHB-inference, representing Known Haplotype Inference on *Beacon database*), respectively (Fig. 1). In the third scenario, we aim to reconstruct a novel haplotype (i.e. that is not observed in public genomes) present in the genome database from the *frequency table* (NHF-Inference).

### 2.1 Known haplotypes inference

We devise a LRT to evaluate if a target haplotype is present in the genome database. Here, we consider the null hypothesis (*H*_0_) and alternative hypothesis (*H*_1_) are:

- *H*_0_: The target haplotype is not in the database.
- *H*_1_: The target haplotype is in the database.

If we can confidently reject the null hypothesis with the *P*-value smaller than a threshold (e.g. 0.05), we may conclude that the target haplotype is present in the private database. i.e. the haplotype is re-identified successfully in the database.

In fact, for a known haplotype *A* (i.e. observed in public human genomes), we can estimate the prior probability (without any query to the database) of its presence in a database *DB* containing *N* genomes (or 2 *N* haplotypes) based on the frequency *f*(*A*) of the haplotype in the population,

(1) $$P(A\in \text{DB})=1-{\left(1-f(A)\right)}^{2N}$$

and

(2) $$P(A\notin \text{DB})=1-P(A\in \text{DB})={\left(1-f(A)\right)}^{2N}.$$

Apparently, for a common haplotype (e.g. f(A)≥0.1), the prior probability may already be high (close to 1.0), indicating for a database of a reasonable size, there is a high probability of at least one genome carries the haplotype. For a minor haplotype, however, the prior probability may not be close to 1.0; the goal of the haplotype-based inference is to increase the confidence using the frequency or Beacon table, if the minor haplotype is indeed present in the database.

Formally, KHF-inference computes the likelihood ratio between the posterior probability ($P(H_{0})$) of a target haplotype present in the genome database and the probability ($P(H_{1})$) of the haplotype not present in the database, both under the condition of the given frequency table of SNVs in the haplotype from the database, thus $P(H_{0})=P(A\in \text{DB}|Q)$ and $P(H_{1})=P(A\notin \text{DB}|Q$). Both probabilities can be estimated from the size of the genome database (*N*), the population frequency of the target haplotype (*f*), and the minor allele frequencies in the *frequency table* or the presence/absence information of the minor alleles in the *Beacon table* using the Bayes’ theorem

P(A∈DB|Q)=

 

$$\frac{P(Q|A\in \text{DB})\times P(A\in \text{DB})}{P(Q|A\in \text{DB})\times P(A\in \text{DB})+P(Q|A\notin \text{DB})\times P(A\notin \text{DB})}$$

 

P(A∉DB|Q)=1−P(A∈DB|Q)

where *Q* represents the frequency or the Beacon table on the SNVs in the haploblock that is either disseminated by the data owner or through beacon queries to the database, P(A∈DB) is the prior probability of the target haplotype *A* being present in the database and P(A∉DB) is the prior probability of the target not being present. The test statistic Λ, i.e. the likelihood ratio, can then be computed by,

(3) $$\Lambda =\frac{P(A\notin \text{DB}|Q)}{P(A\in \text{DB}|Q)}$$

 

(4) $$=\frac{P(Q|A\notin \text{DB})\times P(A\notin \text{DB})}{P(Q|A\in \text{DB})\times P(A\in \text{DB})}$$

 

(5) $$=\frac{P(Q|A\notin \text{DB})\times {\left(1-f(A)\right)}^{2N}}{P(Q|A\in \text{DB})\times (1-{\left(1-f(A)\right)}^{2N})}$$

Because the distribution of the test statistic Λ is unknown, we build the null distribution of Λ by simulating a cohort of human genomes (the *null cohort*) that are not in the genome database to compute the *P*-value and the power of the test. We note that in practice, the null cohort can be constructed by querying the variants in each non-target haplotype observed in public genomes: if any variant in the haplotype is not in the database, this haplotype can be included in the *null cohort*.

The computation of P(Q|A∉DB) and P(Q|A∈DB) is dependent on the information embedded in the summary statistics. Below, we will present algorithms to compute these two conditional probabilities using the *frequency table* and the *Beacon table* as input (*Q*), respectively.

#### 2.1.1 KHF-inference

The input frequency table provides the frequencies (or equivalently the counts) of the minor (or major) allele at each variation site of the haploblock in the database, which imposes the constraints of the haplotypes that may be present in the database. Assuming a haploblock contains a total of *t known* haplotypes (derived from public human genome dataset), our goal is to estimate the frequency of each haplotype *x_i_* in a target private genome database (denoted as $f(x_{i})$). Apparently,

(6) $$\sum_{i=1}^{t}f(x_{i})=1$$

We note that the frequencies of some known haplotypes in the target database may be 0, i.e. no genome in the database carries these haplotypes. We denote $b_{ij}\in \{0,1\}$ as the allele at the *j*th variation site in the *i*th haplotype: *b_ij_* = 0 indicates it is a major allele, while *b_ij_* = 1 indicates it is a minor allele. Then we have,

(7) $$\sum_{i=1}^{t}b_{ij}\times f(x_{i})=f(y_{j})$$

where $f(y_{j})$ is the minor allele frequency at the *j*th variation site in the database, known as the *j*th (j=1,…,L, where *L* is the length of the haploblock) element in the frequency table for the haploblock. The *L* equations in 7 and Equation 6 represent the linear constraints on the frequencies of haplotypes ($f(x_{i})$) in the database.

**Fig. 2.**
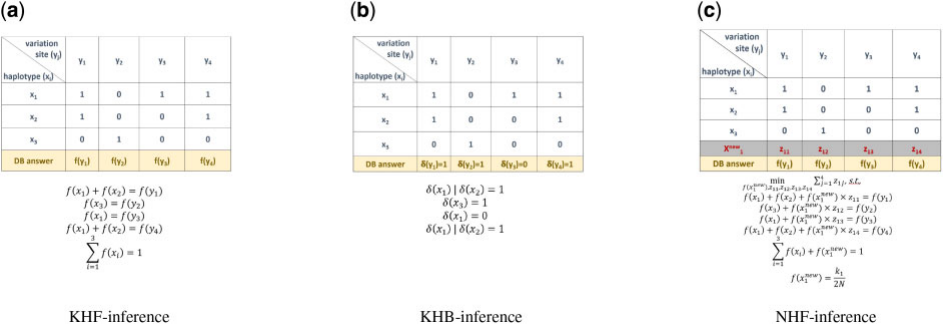
The toy examples of haplotype-based inferences. Three haplotype-based inference algorithms are illustrated by toy examples, including the input tables of allele frequencies (**a** and **c**) or presence/absence (**b**) of the minor alleles at each variation site in a target haploblock, and the linear (a and c) or logical (b) constraints derived from the respective input table

Figure 2 illustrates the linear constraints using a toy example. Consider a haploblock containing *L *=* *4 variation sites (*y*_1_, *y*_2_, *y*_3_ and *y*_4_) and *t *=* *3 known haplotypes (*x*_1_, *x*_2_ and *x*_3_). Assuming the haplotype *x*_1_ contains the minor alleles at *y*_1_, *y*_3_ and *y*_4_, we get $b_{11}=1,\;b_{12}=0,\;b_{13}=1$ and $b_{14}=1$. Similarly, we also know the values of $b_{21},\ldots ,b_{34}$, as shown in Figure 2a. Because the haplotype *x*_1_ and *x*_2_ contain the minor allele at *y*_1_, we can derive the linear *equation* 7 as $f(x_{1})+f(x_{2})=f(y_{1})$, which represents the constraint of the frequencies of *x*_1_ and *x*_2_, given the known minor allele frequency $f(y_{1})$ from the frequency table. Similarly, we can derive a linear constraint for each variation site *y*_2_, *y*_3_ and *y*_4_. In summary, we obtain all the linear constraints of the haplotype frequencies as below,

$$\begin{array}{l} f(x_{1})+f(x_{2})=f(y_{1})\\ f(x_{3})=f(y_{2})\\ f(x_{1})=f(y_{3})\\ f(x_{1})+f(x_{2})=f(y_{4})\\ \sum_{i=1}^{3}f(x_{i})=1 \end{array}$$

These constraints of the linear equations may result in three different outcomes:

- *No solution*. If no solution of $f(x_{i})$ (for i=1,2,…,t) can satisfy all linear equations (in particular when *L* > *t*), we consider a novel haplotype (i.e. that have not been observed in the public genome database) to be present in the target database. We will attempt to reconstruct the sequence ($z_{1},z_{2},\ldots ,z_{L}$) of the novel haplotype using the NHF-inference algorithm presented in Section 2.2.
- *Unique solution*. If there exists a unique solution of $f(x_{i})$ (for i=1,2,…,t) satisfying all linear equations, we then successfully reconstructed the haplotypes of this haploblock in the target database given the input frequency table. A haplotype *A* is present if and only if *f*(*A*) > 0, and thus P(A∈DB|Q)=1, P(Q|A∈DB)=1 and P(Q|A∉DB)=0. Otherwise, the haplotype *A* is not present in the target database, i.e. P(A∉DB|Q)=1, P(Q|A∈DB)=0, P(Q|A∉DB)=1.
- *Multiple solutions*. If multiple solutions exist in the linear equations, the probability of observing the frequency table under the condition that a haplotype *A* is present in the database (P(Q|A∈DB)) can be computed by the sum of the probabilities of all solutions containing the haplotype *A*, whereas the probability of observing the frequency table under the condition that the haplotype *A* is not present in the database (P(Q|A∉DB)) can be computed as the sum of the probabilities of all solutions not containing the haplotype *A*. Here, a solution of the linear equations gives the frequencies of all *t* known haplotypes, $f(x_{i})$ for i=1,2,…,t. Therefore, we have
  (8) $$P(Q|A\in DB)=\sum_{k=1}^{m}b_{k}\left(\begin{array}{c} 2N\\ n_{k} \end{array}\right)f^{k}{\left(A\right)}^{n_{k}}{\left(1-f^{k}(A)\right)}^{2N-n_{k}}$$
   
  (9) $$P(Q|A\notin DB)=\sum_{k=1}^{m}\left(1-b_{k}\right){\left(1-f^{k}(A)\right)}^{2N}$$

where *m* is the number of solutions of the linear equations, $f^{k}(A)$ is the frequency of the haplotype *A* in the *k*th solution, *n_k_* is the count of haplotype *A* in the *k*th solution, i.e. $n_{k}=2Nf^{k}(A)$, and $b_{k}\in \{0,1\}$: *b_k_* = 1 when $n_{k}>0$, and *b_k_* = 0 when *n_k_* = 0.

In practice, we use the python package sympy.solve (Meurer et al., 2017) to solve the linear equations derived from the frequency table, and compute the likelihoods and the test statistic accordingly.

#### 2.1.2 KHB-inference

We use a similar LRT for the membership inference given the Beacon table from a target genome database. Intuitively, the Beacon table offers the information if a minor allele is present or absent in the database, from which we may define a set of logical constraints about the presence/absence of a haplotype in the database. For examples, if a minor allele *b* is reported to be present in the database:

1. if only one haplotype in the haploblock contains the minor allele *b*, this haplotype must be present in the database.
2. if two or more haplotypes contain *b*, at least one of these haplotypes must be present in the database.
3. no constraint is imposed on the other haplotypes not containing b.

On the other hand, if a minor allele *b* is not present in the database, then

1. all haplotypes containing *b* cannot be present in the database.
2. no constraint is imposed on the other haplotypes not containing b.

It is obvious the logical constraints are not as stringent as the linear equations derived from the frequency table as presented above. However, they also improve the posterior probability estimation of the haplotypes to be present in the database.

Figure 2b illustrates the logical constraints using a toy example. Similar to Figure 2a, we consider a haploblock containing *L *=* *4 variation sites and *t *=* *3 haplotypes. Assume we know the minor allele at the variation site *y*_1_ is present in the database. It implies that at least one of the haplotypes *x*_1_ and *x*_2_ must be present in the database, whereas the haplotype *x*_3_ may or may not be present. If we further know that the minor allele at the site *y*_2_ is present in the database, then we deduce the haplotype *x*_3_ must be in the database. Finally, if we also know that the minor allele at the site *y*_3_ is not present in the database, then the haplotype *x*_1_ cannot be present in the database. At *y*_4_, haplotype *x*_1_ and *x*_2_ have the minor allele, which is the same situation as of site *y*_1_. Thus, the presence of *y*_4_ does not provide additional linear constraint to haplotypes $x_{1},x_{2},x_{3}$. Taking these constraints together, we can conclude the haplotypes *x*_2_ and *x*_3_ must be present in the database, while *x*_1_ is not present.

The haploblock structure in the human genome is more complex than the toy example shown above. In practice, even though the presence of some haplotypes may not be fully determined based on the logical constraints, we may still get a better estimate of the probability for them to be present in the database. In this case, similar to the approach adopted in KHF-inference algorithm, we aim to estimate P(Q|A∈DB) based on the putative haplotype compositions satisfying the logical constraints. Consider a haploblock containing a total of *t* putative haplotypes. The counts of different haplotypes in the database then follow a multinomial distribution with the sum of counts =2N. When logical constraints are applied, we may approximate P(Q|A∈DB) using the cumulative density function (*cdf*) of the multinomial distribution . For example, in a haploblock containing 3 haplotypes (*x*_1_, *x*_2_ and *x*_3_), based on the logical constraints, we know that at least one of *x*_1_ and *x*_2_ are present in the database, while no constraint is applied to *x*_3_. As a result, P(Q|A∈DB) can be computed as,

$$\begin{array}{l} P(Q|x_{1}\in DB)=p(n_{1}\geq 1,n_{2}\leq 2N,n_{3}\leq 2N)\\ P(Q|x_{1}\notin DB)=p(n_{1}=0,n_{2}\geq 1,n_{3}\leq 2N)\\ P(Q|x_{2}\in DB)=p(n_{1}\leq 2N,n_{2}\geq 1,n_{3}\leq 2N)\\ P(Q|x_{2}\notin DB)=p(n_{1}\geq 1,n_{2}=0,n_{3}\leq 2N)\\ P(Q|x_{3}\in DB)=p(n_{1}\leq 2N,n_{2}\leq 2N,n_{3}\geq 1)\\ -p(n_{1}=0,n_{2}=0,n_{3}\geq 1)\\ P(Q|x_{3}\notin DB)=p(n_{1}\leq 2N,n_{2}\leq 2N,n_{3}=0)\\ -p(n_{1}=0,n_{2}=0,n_{3}=0) \end{array}$$

where *p* represents the *cdf* of multinomial distribution with the counts of three haplotypes as *n*_1_, *n*_2_ and *n*_3_, respectively, and $n_{1}+n_{2}+n_{3}=2N$.

In practice, because the computation of the *cdf* of multinomial distribution is quite tedious, we approximate the value of multinomial *cdfs* by sampling the multinomial distribution according to the estimated frequency (from public resource) of each known haplotype. The python solver numpy.random.multinomial (Harris, 2020) is applied to build the multinomial sample sets (5000 data points in each sample set). This approximation method significantly accelerates the computation of the *cdf* of the multinomial distribution, which takes only a few seconds to compute one *cdf* in a single CPU.

**

### 2.2 Reconstruction of novel haplotypes and NNF-inference

As discussed in Section 2.1.1, when no solution can satisfy all linear equations imposed by the input frequency table, we hypothesize one or more *novel* haplotype are present in the target database. A novel haplotype is the one that is not observed in the public genome database, and thus is not considered among the *t* haplotypes when we devise the constraints.

Here, we attempt to reconstruct the novel haplotype (i.e. the allele at each variation site in the haploblock) using an integer linear programming (ILP) approach. Below, we incorporate *d* types of novel haplotypes denoted as $x_{1}^{\mathrm{new}},\ldots ,x_{d}^{\mathrm{new}}$ in addition to the *t* known haplotypes into the linear constraints:

(10) $$\sum_{i=1}^{t}b_{ij}\times f(x_{i})+\sum_{m=1}^{d}f(x_{m}^{\text{new}})\times z_{mj}=f(y_{j})$$

(11) $$\sum_{i=1}^{t}f(x_{i})+\sum_{m=1}^{d}f(x_{m}^{\text{new}})=1$$

(12) $$f(x_{m}^{\text{new}})=\frac{k_{m}}{2N},m\in [1,d]$$

where $x_{i},i=1,2,\ldots ,t$ represent the *t* known haplotypes, *z_mj_* represents the (unknown) allele at the *j*th site of the *m*th novel haplotype, i.e. *z_mj_* = 0 if $x_{m}^{\mathrm{new}}$ has a major allele at the *j*th site, and *z_mj_* = 1, otherwise, and $f(x_{m}^{\text{new}})$ is frequency of the *m*th novel haplotype. *k_m_* is the count of the *m*th novel haplotype. The other notations follow those used in Section 2.1.1. However, different from the goal in the KHF-inference, here we want to solve the unknown variables *z_mj_* in addition to the haplotype frequencies ($f(x_{m}^{\text{new}})$ and $f(x_{i})$) using these linear constraints.

We may extend the example used in Section 2.1.1 to illustrate NHF-inference algorithm. Here, we assume *d* novel haplotype variants $x_{1}^{\mathrm{new}},\ldots ,x_{d}^{\mathrm{new}}$ with unknown alleles in addition to the 3 known haplotype variants $x_{1},x_{2},x_{3}$ are potentially present in the database, as shown in Figure 2c. Intuitively, because the novel haplotypes are not observed in the public genome database, it is likely a rare combination of alleles, but should still contain a majority of major alleles. Hence, we set the objective function of the ILP problem as to minimize $\sum_{m=1}^{d}\sum_{j=1}^{3}z_{mj}$ (i.e. to minimize the total number of minor alleles in all *d* novel haplotype variants), subject to the linear constraints imposed by the frequency table. Initially, we set the number novel haplotype variants as 1 (i.e. *d *=* *1).

$$\begin{array}{l} min_{f(x_{1}),f(x_{2}),f(x_{3}),f(x_{1}^{\mathrm{new}}),z_{11},z_{12},z_{13},z_{14}}\sum_{j=1}^{l=4}z_{1j}\;\;\;\;\;s.t.\\ f(x_{1})+f(x_{2})+f(x_{1}^{\mathrm{new}})\times z_{1}=f(y_{1})\\ f(x_{3})+f(x_{1}^{\mathrm{new}})\times z_{2}=f(y_{2})\\ f(x_{1})+f(x_{1}^{\mathrm{new}})\times z_{3}=f(y_{3})\\ f(x_{1})+f(x_{2})+f(x_{1}^{\mathrm{new}})\times z_{4}=f(y_{4})\\ \sum_{i=1}^{3}f(x_{i})+f(x_{1}^{\mathrm{new}})=1\\ f(x_{1}^{\mathrm{new}})=\frac{k_{1}}{2N} \end{array}$$

In practice, we run multiple times of the pulp. LpProblem ILP solver (Mitchell et al., 2011), each time with the increasing *k*_1_, i.e. the count of the novel haplotype in the database, from 1 to 2 *N*. For the smallest *k*_1_ value leading to a solution (note that when $k_{1}=0$, there is no solution) of the ILP problem, we will consider the corresponding *z*_1_, *z*_2_ and *z*_3_ as the alleles of the putative novel haplotype, and $f(x_{1}^{\mathrm{new}})$ as its frequency in the target database.

Once the novel haplotype variants *x^new^* are reconstructed, we further perform the KHF-inference algorithm to compute the confidence of the haplotype variants to be present in the database based on the LRT (see Section 2.1.1 for details). Starting from *d *=* *1, we increase the value of *d* (i.e. the number of types of novel haplotype variants) by 1 if no solution found and perform ILP again until *d* is greater than a threshold (e.g. 2). The complexity of this algorithm is $O({\left(2N\right)}^{d})$, which is feasible for only small *d*.

## 3 Experiments and results

We implement the three haplotype-based membership inference algorithms in Python 2.7 (Van Rossum and Drake, 1995) [utilizing the packages sympy.solve (Meurer *et al.*, 2017) and pulp. LpProblem (Mitchell *et al.*, 2011)], and evaluate them using an artificially created target genome database comprising 500 randomly selected human genomes (i.e. 1000 haplotypes) from the 1000 Genomes Project data (GenomeAsia100K Consortium, 2015). We consider the entire set of 2147 genomes in the 1000 Genomes Project (GenomeAsia100K Consortium, 2015) as the public genome database, from which we derive the haploblocks as well as haplotypes in each block in the chromosome 10 in human genome using HaploView (Barrett *et al.*, 2005). Afterwards, we use each *minor* haplotype, i.e. with a low frequency in the public database such that its prior probability of being present in the target database is <0.1 (even though it is present), as a target haplotype, and attempt to test if it is present in the database. When evaluating the KHF-inference and the KHB-inference algorithms, we consider all haplotypes in the target haploblock (i.e. the same halpoblock as the target haplotype) that are derived from the public database as the *known* haplotypes. To evaluate the performance of the NHF-inference algorithm, we exclude one (or two) haplotype in the target haploblock, and attempt to reconstruct it using the frequency table computed from the target genome database. We note that even though the experiments are successful for many testing cases, for some target haplotypes, the inference algorithms (especially for the KHB-inference algorithm) cannot finish in a reasonable amount of time, and thus are terminated (see below for details). It may be due to several different reasons: the solver for the linear equations is slow for KHF-inference, or the pulp. LpProblem ILP solver cannot reach a solution for NHF-inference.

### 3.1 Construction of haploblocks and haplotypes

We use Haploview (Barrett *et al.*, 2005) to derive the haploblocks as well as the haplotypes in each block from the chromosome 10 of the 2147 human genomes in the public database (i.e. from the 1000 Genomes project). Because we want to enhance the confidence of the presence of a rare haplotype in a target genome database, we select only the haplotypes that are rare in the pubilc database (i.e. those with small prior probability P(A∈DB)<0.1), and contain 50 or more variation sites (i.e. L≥50) as the putative target haplotypes. In total, we select 1350 target haplotypes on 100 haploblocks based on the above criteria. Each target haploblock contains up to 187 variation sites and 55 haplotypes. We perform KHF-interence and KHB-inference algorithms on these haplotypes.

In addition, we select another 484 haploblocks (which may be overlapped with the above 665 haploblocks) from the chromosome 10 that contain at least one haplotype with the frequency smaller than 0.02 to evaluate the NHF-inference algorithm, in which we first remove the least frequent haplotype and then attempt to reconstruct it.

### 3.2 Re-identification of known haplotypes

To perform the KHF-inference and KHB-inference algorithms, we first compute the empirical distribution of the LRT test statistic Λ under the null hypothesis. We construct a dataset containing 276 haplotypes on the chromosome 3 of 100 genomes from the 1000 Genomes Project (GenomeAsia100K Consortium, 2015), and then compute the test statistic Λ on this dataset, which is used as the empirical null distribution of Λ, when evaluating the confidences in the KHF-inference and KHB-inference algorithms.

Next we attempt to test if each of the 1350 target haplotypes is present in the target database using KHF-inference and KHB-inference algorithms, respectively. The *P*-value of the LRT is calculated based on the test statistic Λ and its null distribution. We compare the power of the haplotype-based inference with the SNV-based inference methods: the KHF-inference is compared with the LRT, an improved SNV-based inference method than the test statistic proposed by Homer *et al.* (2008) and is shown to be near-optimal (Sankararaman *et al.*, 2009; Wang *et al.*, 2009), while the KHB-inference is compared with the optimal attack (Raisaro *et al.*, 2017), which shows better performance than the original method proposed by Shringarpure and Bustamante on Beacon tables (Shringarpure and Bustamante, 2015). The SNV-based inference methods are performed by using the allele at each variation site of the target haplotype in the respective test statistics. The powers of both the haplotype-based and the SNV-based inference methods are measured by using the fraction of haplotypes in each haploblock can be detected with high confidence (i.e. *P*-value < 0.05).

**Fig. 3.**
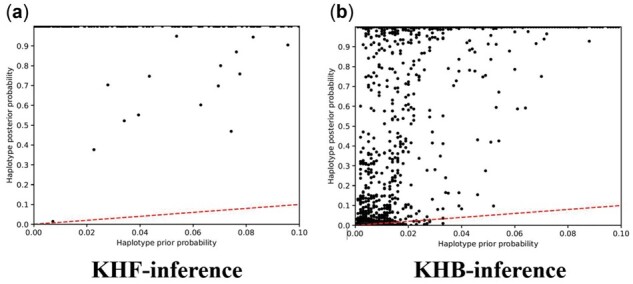
The comparison between the posterior (*y*-axis) and the prior (*x*-axis) probabilities of target haplotypes before and after applying the KHF-inference on the frequency table (left) and the KHB-inference on the Beacon table (right). Each target haplotype is represented by a dot, and the red dashed lines represent the diagonal, indicating equal values of the posterior and prior probabilities. Particularly, in KHF-inference, we obtain the posterior probability equals 1.0 on 475 haplotypes, which is the outcome of a unique solution received from the linear equations

We first show that the posterior probability of the target haplotypes to be present in the target database (P(A∈DB|Q)) computed by the KHF-inference and KHB-inference algorithms are significantly higher than the corresponding prior probabilities (P(A∈DB)) of the same haplotypes, which demonstrates the Bayesian methods indeed retrieve the information leaked in the frequency table and Beacon table. Figure 3 compares the prior and posterior probabilities of all the haplotypes that complete the LRT (each represented by one dot in the figure) by using the KHF-inference (Fig. 3a) and KHB-inference (Fig. 3b). The lines in the figure show the diagonal, indicating equal values of prior and posterior probabilities. Note that all of the tested haplotypes are present in the target database. As a result, we observe that the posterior probabilities of all tested haplotypes are much higher than their prior probabilities; 486 (36.4%) haplotypes receive the posterior probabilities above 0.9 in the KHF-inference, although their prior probabilities are all below 0.1. Specifically, we receive a unique solution from the linear equations on 475 haplotypes from 32 haploblocks, which implies that the posterior probabilities of these haplotypes reach 1.0 according to Section 2.1.1. The improvement of the KHB-inference algorithm is also considerable, even though the Beacon table is considered to leak less information. The posterior probabilities of some haplotypes are substantially improved, including 520 (38.5%) haplotypes receive the posterior probabilities above 0.9, indicating the logical constraints imposed by the presence/absence of specific allele may lead the re-identification of these haplotypes. In addition, we further construct 297 hapblotypes that are not in the target database with prior probability < 0.1 from chromosome 2. The KHF-inference and KHB-inference methods report 29 and 28 haplotypes, respectively, to be in the target database (with *P*-value < 0.05), indicating the false positive rate is about 10% as expected. The haplotype-based inference algorithms show greater power than conventional SNV-based inference methods, as demonstrated below.

#### 3.2.1 KHF-inference

The KHF-inference algorithm successfully finishes on 503 (37.3%) out of 1350 target haplotypes over 89 haploblocks, among which 503 haplotypes (100%) receive *P*-value (estimated by the null-distribution) smaller than 0.05.

**Fig. 4.**
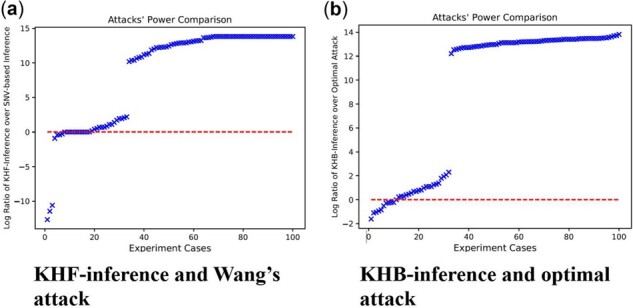
The comparison of the statistical power, i.e. the fraction of confidently identified target haplotypes (with *P*-value < 0.05 based on the likelihood ratio test) between the haplotype-based inference methods and SNV-based inference methods. Left: the log ratio between the power of the KHF-inference and that of the SNV-based likelihood ratio test on the frequency tables; and right: the log ratio between the KHB-inference and the optimal attack on the Beacon tables. The power is evaluated on each tested haploblock, represented by one point in the figure, sorted based on the log ratio of the two powers. The red dashed lines indicate the cases when the haplotype-based and SNV-based inferences achieved the same re-identification power.)

Compared to the LRT test statistic for SNV-based inference (Wang *et al.*, 2009), the KHF-inference is significantly more powerful on the same target haploblocks. Figure 4a shows the log ratio between the power of these two inference methods: the power of KHF-inference is greater than that of SNV-based inference on 82 out of 100 target haploblocks (82% experiment cases), each represented by one point in the figure sorted based on the log ratio between their power. The maximum log ratio between the power of KHF-attack and that of the SNV-based inference is 13.82, i.e. the power of KHF-attack is 100% (successfully infer all the target haplotypes in a block) while the power of near-optimal SNV-based inference is approximately 0.0% (fail to infer any target haplotype in a block).

#### 3.2.2 KHB-inference

The KHB-inference algorithm successfully finishes on 1350 (100%) out of 1350 target haplotypes over 100 haploblocks. Among these target haplotypes, 644 haplotypes (47.7%) receive *P*-values smaller than 0.05.

Compared to the optimal attack (Raisaro *et al.*, 2017), KHB-inference is much more powerful on the same haploblock. Figure 4b shows the log ratio between the power of these two inference methods: the power of KHB-inference is greater than that of the optimal attack (Raisaro *et al.*, 2017) on 89 out of 100 haploblocks (89% experiment cases), each represented by one point in the figure sorted based on the log ratio between their power. The maximum log ratio between the power of KHB-inference over the optimal attack is 13.82, i.e. the power of KHB-inference is 100% (successfully infer all the target haplotypes in a block) while the power of the optimal attack on the Beacon database is approximately 0.0% (fail to infer any target haplotype in a block).

### 3.3 Reconstruction of novel haplotypes

We evaluate the NHF-inference on the *frequency table* from the target database on each of 484 haploblocks that contains at least haplotype with frequency smaller than 0.02. The workflow of the experiment is as following:

1. Pre-process the database by removing the genomes that contain the haplotype with smallest frequency in each haploblock (i.e. considered as the novel haplotype.
2. Perform the NHF-inference algorithm on the processed database, and discard the haploblocks from which the removed (novel) haplotypes cannot be reconstructed.
3. Perform KHF-inference algorithm on the reconstructed novel haplotypes from *Step 2* and report the ones with *P*-value smaller than 0.05.

In our experiment, we successfully reconstruct 406 (out of 484) novel haplotypes by NHF-inference, among which 161 (33.3%) are reported to be *present in the target database* by the KHF-inference algorithm (with *P*-value < 0.05). Comparing the the ground truth (the original haplotypes removed in the *Step 1*), all the 161 (100%) reconstructed novel haplotypes are completely correct, implying the objective of the ILP formulation that minimizes the number of minor alleles in the novel haplotype is reasonable. The average length of these correctly reconstructed haplotypes is 24.2. Their lengths vary significantly (standard deviation =25.5), which implies that the length of the novel haplotype seems not to affect the success rate of the reconstruction algorithm. On the other hand, the average count of the correctly reconstructed novel haplotypes in the database is 10 (standard deviation =1), the same as the average count of all 484 novel haplotypes, which indicates that some novel haplotypes with low frequencies can be successfully constructed. Similarly, we perform NHF-inference algorithm on 448 haploblocks by removing two (novel) haplotypes. We are able to reconstruct both of the two novel haplotypes on 353 hapbloblocks by using NHF-inference. By applying the KHF-inference algorithm, on 124 (27.7%) haploblocks, both of novel haplotype variants are reported as *in the target database* (with *P*-value < 0.05), among which 124 (100%) cases are correctly reconstructed. We note that it becomes more difficult to reconstruct the novel haplotypes if the target database contains more than one novel haplotype variants. However, the power of this method will increase with more human genomes become available in public, in which more haplotype variants will be covered.

## 4 Conclusions and discussions

In this article, we propose three haplotype-based membership inference algorithms for inferring the presence of rare haplotypes (with very low frequency in the general population) in a target private genome database using the allele frequencies or the presence/absence of minor alleles that are shared by the data owners. These methods do not require the target’s genome, and thus may represent a new kind of privacy risk in genomic data sharing. The re-construction of a rare haplotype may achieve much stronger power than one or more rare individual SNPs. Specifically, we can reconstruct a rare haplotype variant consisting of 50 or more SNPs (with the estimated frequency smaller than 0.02 in the general population) using the NHF-inference algorithm. Such a rare haplotype variant alone is almost sufficient to identify a human subject (or its close blood relatives) in the cohort.

Based on these results, several linking attacks can be implemented to infer private information about participants in genomic studies without the access of any genetic data from the red target. For examples, if one or more rare haplotype variant is constructed from two different genomic databases, one may infer the same human subject (or two close relatives) participated in these two corresponding cohorts. Furthermore, with appropriate statistical analyses, it may even be inferred that the rare haplotype variants shared by the two datasets are likely from a single human subject, thus establish a powerful linkage among rare haplotype variants. In addition, the reconstructed rare haplotype variant may be known to be associated with a rare phenotype [e.g. a Denisovan haplotype was recently discovered to be associated with the lip thickness (Bonfante et al., 2021)], in which case the phenotypes (e.g. the facial appearance) of the human subject can be inferred. Finally, the reconstructed rare haplotype variants may be used to infer the ethnic or even family origin of a target human subject because sharing a rare, long haplotype often indicate the likelihood of common ancestor (Kong et al., 2008).

On the other hand, we show that, when the target’s genome is available, the haplotype-based inference algorithms achieve greater re-identification power compared to the SNV-based inference methods. These results suggest the current defense strategies that are mostly focused on mitigating the SNV-based inference methods (Ayoz *et al.*, 2020b; Bu *et al.*, 2018; Raisaro *et al.*, 2017) may not be sufficient to fully eliminate the risks in genomic data sharing. In the future, we plan to develop novel defense methods to reduce the privacy risks in human genomic data sharing due to haplotype-based membership inference, while maintaining the utility of shared data from human genome databases.

We note that our current problem formulation does not consider *de novo* mutations or sequencing errors that may occur at the SNV sites in the target private database. Even though the chance is extremely low, their occurrences will result in novel haplotype variants, which may be reconstructed by using the NHF-inference method. Afterwards, the mutation or sequencing error can be easily detected, because these artificial variants differ from a common haplotype at only one SNV. Manual inspection of the raw sequencing data is then needed in order to determine if it is indeed a *de novo* mutation.

## Funding

The research was partially supported by the National Institute of Health [U01EB023685 and R01HG010798], National Science Foundation [CNS-1838083] and Indiana University (IU) Precision Health Initiative (PHI).

*Conflict of Interest*: none declared.
